# Association of MMP-2 gene haplotypes with thoracic aortic dissection in chinese han population

**DOI:** 10.1186/s12872-016-0188-4

**Published:** 2016-01-14

**Authors:** Ou Liu, Jiachen Li, Yi Xin, Yanwen Qin, Haiyang Li, Ming Gong, Yuyong Liu, Xiaolong Wang, Jianrong Li, Hongjia Zhang

**Affiliations:** Department of Cardiovascular Surgery, Beijing An Zhen Hospital, Capital Medical University, Beijing, 100029 China; Beijing Institute of Heart Lung and Blood Vessel Diseases, Beijing, 100029 China

**Keywords:** MMP-2 gene, Single nucleotide polymorphism, Thoracic aortic dissection

## Abstract

**Background:**

Thoracic aortic dissection (TAD) is the most common life-threatening disorder, and MMP-2 is involved in TAD pathogenesis. Our purpose is to systematically evaluate the association of the MMP-2 gene with TAD risk in Chinese Han population.

**Methods:**

In our case–control study, we recruited 755 unrelated participants: 315 case participants with TAD and 440 controls. Twenty-two tag SNPs were selected from MMP-2 gene and were genotyped. Genotype data were analyzed by logistic regression.

**Results:**

Although we did not find any significant association for MMP-2 SNPs using single-marker analysis, we identified many windows with haplotype frequencies significantly different between case participants and control participants using a variable-sized sliding-window strategy. In particular, the most significant association was shown by a 2-SNP window consisting of rs2241145 and rs9928731 (omnibus test: asymptotic P_asym_ = 7.48 × 10 ^−5^ and empirical P_emp_ = 0.001867). There were two protective haplotypes: CT (P_asym_ = 0.00303; odds ratio [OR], 0.403) and GC (P_asym_ = 0.000976; OR, 0.448).

**Conclusions:**

MMP-2 haplotypes are associated with genetic susceptibility to thoracic aortic dissection in Chinese Han population.

**Electronic supplementary material:**

The online version of this article (doi:10.1186/s12872-016-0188-4) contains supplementary material, which is available to authorized users.

## Background

Thoracic Aortic dissection (TAD) is a complex cardiovascular disease with high morbidity and mortality [1]. It can be affected by both genetic and environmental factors as well as gene environment interaction [2]. It is believed that hypertension is the main etiological risk factor in aortic dissection development [3]. Clinical and genomic evidence also shows that genetics plays an important role in the development of thoracic aortic diseases [4, 5]. However, the exact mechanism underlying thoracic aortic dissection is still unclear [6, 7].

Inherited tissue connective diseases and atherosclerosis are the main diseases related to TAD [8]. It is well known that MMPs are deeply involved in the pathogenesis of both inherited and not-inherited conditions [8]. For example, MMP-12 in particular has not only been related to dissection occurring in thoracic aorta of subjects who were not affected by inherited diseases [9, 10], but also in patients with Marfan syndrome [11]. Although in some inherited TAD patients a specific pattern of MMPs/TIMPs has been identified, it is still unclear in TAD patients with atherosclerosis [8]. A widespread release of MMPs, such as MMP-1, −2, −3, −8, −9,and −12 have been reported in patients with aortic wall diseases and no genetic predisposition [8]. In this field, MMP-2 has been studied intensively [8, 12]. It has been shown that the expression level of MMP-2 in the aortic walls was significantly higher in the TAD than the normal group [7]. MMP-2 is one of tightly regulated family of zinc dependent enzymes which is important in extracellular matrix (ECM) degradation and remodeling [13]. It has been demonstrated that MMP-2 is involved in pathogenesis of some tissue remodeling-related diseases where the single nucleotide polymorphisms (SNPs) were found significantly associated with these diseases [14–16]. A haplotype with both rs11644561 A and rs11643630 G in MMP-2 gene was found to have a significantly reduced risk of breast cancer (OR, 0.6; 95 % CI, 0.4–0.8) in a study of 6066 participants carried out by Vanderbilt-Ingram Cancer Center [14]. Another several MMP-2 single nucleotide polymorphisms (SNPs) have been identified to be associated with the pathogenesis of some other tissue remodeling-related diseases, such as systolic heart failure and stroke [15, 16]. Genetic evidence supporting a role for MMP-2 in tissue remodeling-related diseases has come from these analyses.

Aortic dissection is also one of tissue remodeling-related diseases [17]. The histological appearance of aortic dissection is characterized by progressive degradation of extracellular matrix proteins by some proteolytic enzymes, e.g. MMP-2 [18]. All these findings strongly suggest that MMP-2 may play a specific role in the development of TAD among the whole matrix metalloproteinase family. Although a number of studies have identified a link between the MMP-2 and the development of thoracic aortic dissection [19, 20], none have used a genetic approach to evaluate allelic variation in MMP-2 and odds of thoracic aortic dissection, especially for not-inherited condition. So we aimed to assess the association between MMP-2 gene and not-inherited TAD.

The implementation of the International HapMap Project has enabled rapid acquisition of data on common SNPs in an entire gene and exploration of disease-associated genetic variants in that gene using a comprehensive approach [21]. In the current work, a tag SNP approach was employed to probe common genetic variations in the MMP-2 gene as well as to construct haplotype blocks where appropriate to determine the role of this gene in the development of not-inherited thoracic aortic dissection.

## Methods

### Patient recruitment

We enrolled 315 patients with TAD referred to the Cardiovascular Surgery Unit. The criterion for diagnostics of TAD has been described previously [22]. Familial TAD were excluded from the study. Familial TAD was defined when one or more family members was affected by TAD. Four hundred forty volunteers comparable for age and ethnicity were used as controls. All controls were selected from individuals who were admitted to Beijing An Zhen Hospital for reasons other than aortic diseases, mainly primary hypertension disease. All controls had a negative history of vascular diseases. Patients and controls were unrelated. All subjects underwent at least one type of aortic imaging examination, including CT scan, MRI, echocardiography, and angiography. Study participants were interviewed in person by trained medical professionals using a structured questionnaire. Detailed information on demographic factors, prior disease history, tobacco and alcohol use, diet, weight history, and family history of aortic disease were collected for all participants. All protocols involving human specimens were approved by the Institutional Review Board at Beijing An Zhen Hospital. Each subject provided written informed consent.

### Blood sample collection and genomic DNA extraction

Ethylenediamine tetraacetic acid (EDTA) anticoagulated venous whole blood samples were collected from each participant. Human genomic DNA was extracted using the DNA Isolation Kit (Genomic DNA kit, Axygen Scientific Inc, CA, USA) according to manufacturers’ instruction. Laboratory staffs were blinded to the case–control status of these subjects for all subsequent genotyping described.

### SNP selection and genotyping

The tagger SNPs were selected by searching Han Chinese data from the HapMap Project using the Tagger program in Haploview 4.2 (Broad Institute, Cambridge, Massachusetts, USA). Tag SNPs were identified to cover polymorphisms with minimum minor allele frequency (MAF) of 0.05 or greater in the MMP-2 gene and with an r^2^ of 0.80 or greater. Selection of SNPs was completed in November 2009. In addition to this tag SNP selection strategy, several high-interest SNPs that have been reported in the literature were also chosen. In this study, a total of 22 SNPs were identified.

Genotyping analysis of the SNPs was performed using the MassARRAY platform (Sequenom, San Diego, CA, USA). The chip-based matrix-assisted laser desorption ionization time-of-flight (MALDI-TOF) mass spectrometry technology is used in this procedure. Genotyping quality was assessed by examination of duplicate concordance and call rates for each SNP and a test for compliance with Hardy-Weinberg equilibrium (HWE) in controls.

### Statistical analysis

Statistical analyses were performed on computer using the statistical genetics software packages PLINK (version 1.07) and Haploview (version 4.2). Hardy–Weinberg equilibrium (HWE) for genotypic distribution was determined by using the Pearson *χ*2 test for each group. Group differences in demographic and baseline clinical data were compared using Pearson *χ*^2^ test in case of qualitative data and with t-tests for independent samples in case of quantitative data. Comparisons of the distributions of the genotype, allele and haplotype frequencies were performed using the Pearson *χ*^2^ test. Bonferroni corrections for multiple comparisons were performed. In order to detect informative associations complementary to our tagging SNP approach, haplotype analysis was performed. Linkage disequilibrium (LD) blocks were constructed among the SNPs based on the default algorithm of Gabriel et al. [23]. Moreover, the sliding window approach was chosen to systematically analyze all possible haplotypes. We performed an omnibus test of the haplotype association to jointly evaluate the significance of the haplotype effects for sliding windows. Sliding windows analyses assess the frequency of composite genotypes of a fixed number of contiguous SNPs (shifting 1 SNP at a time). We used 15,000 random permutations to control for false-positive findings.

## Results

### Study participants

Baseline and clinical characteristics of the subjects are shown in Table 1. As expected, there were significantly more male than female TAD patients included in this study. We found statistical significant gender difference in the occurrence of TAD. There were no significant differences in the proportion of smoking habit, alcohol abuse and other cardiovascular risk factors between TAD cases and controls.

**Table 1:** Demographic and clinical characteristics of both TAD cases and healthy controls in a Chinese Han Population

Items	Controls (n = 440)	TAD patients (n = 315)	p Value
Age	51.3 ± 14.4	51.3 ± 11.1	NS
Gender (male) n (%)	223 (50.7)	241 (76.5)	<0.01
Smoking n (%)	191 (43.4)	153 (48.6)	NS
Alcohol n (%)	92 (20.9)	75 (23.8)	NS
Diabetes n (%)	65 (14.8)	53 (16.8)	NS
Hypertension n (%)	367 (83.4)	258 (81.9)	NS
Dyslipidaemia n (%)	258 (58.6)	198 (62.9)	NS
CAD n (%)	73 (16.6)	68 (21.6)	NS

*Abbreviations*: *TAD* thoracic aortic dissection, *CAD*coronary artery disease, *NS* not significant. Continuous data were tested using 2-tailed Student *t* test and categorical data were tested using a Pearson *χ* ^2^ test (with df = 1) for differences between TAD (patient) and control (normal) groups

### Genetic association analysis

The observed genotype distributions for all SNPs were in Hardy-Weinberg equilibrium in the control group and in the case group. In Table 2, the allele frequencies and genotype distributions of all SNPs and the Pearson *χ*^2^ analysis were reported. The strongest association was observed at the rs11644561 coding variant, where the frequency of the risk allele A was 13.02 % in TAD subjects compared with 9.78 % in controls (P = 0.04813). However, after correction for multiple testing, we found no significant association between this SNP and the TAD phenotype (data not shown). In summary, we did not find a significant association with TAD for all 22 SNPs under study using single marker analysis.

**Table 2:** Allelic Association Tests for Single-Nucleotide Polymorphisms of the MMP-2 Gene

SNP	Allelesa Genotype (1/11/22/2)^a^			P (genotypic)	Minor Allele Frequency		P (allelic)
	1/2	Cases	Controls		Cases	Controls	
rs11644561	A/G	8/66/241	7/72/361	0.1574	0.1302	0.0978	0.04813
rs11643630	G/T	51/148/113	81/226/131	0.1918	0.4006	0.4429	0.1026
rs243866	A/G	2/62/251	3/88/348	.09746	0.1048	0.1071	0.8863
rs243865	T/C	2/61/252	3/87/350	0.9744	0.1032	0.1057	0.8753
rs17859821	A/G	20/127/167	21/167/251	0.4407	0.2659	0.2380	0.2177
rs1030868	T/C	31/123/161	41/176/221	0.9337	0.2937	0.2945	0.9709
rs1477017	G/A	31/123/161	41/175/222	0.9514	0.2937	0.2934	0.9909
rs865094	G/A	24/129/162	43/180/215	0.5559	0.2810	0.3037	0.3402
rs17301608	T/C	49/143/123	71/211/154	0.5848	0.3825	0.4048	0.3836
rs1053605	T/C	7/76/232	4/101/335	0.3234	0.1429	0.1239	0.282
rs9302671	T/G	10/90/214	16/136/287	0.7480	0.1752	0.1913	0.4247
rs2241145	G/C	76/154/84	107/212/121	0.9667	0.4873	0.4841	0.9034
rs9928731	T/C	75/147/92	93/219/128	0.6140	0.4729	0.4602	0.6259
rs243849	T/C	13/98/204	14/123/294	0.7159	0.1968	0.1818	0.4618
rs243847	C/T	41/156/117	75/215/147	0.2625	0.3790	0.4176	0.139
rs2287074	A/G	30/132/150	43/176/215	0.8832	0.3077	0.3011	0.7863
rs1992116	T/C	30/134/151	43/176/220	1.7959	0.3079	0.2984	0.691
rs243839	G/A	47/157/110	69/217/151	0.9635	0.3997	0.4062	0.8001
rs11639960	G/A	32/126/157	39/185/210	0.7226	0.3016	0.3030	0.9533
rs243836	G/A	51/160/96	76/211/149	0.5814	0.4395	0.4163	0.3699
rs243835	C/T	44/150/120	54/224/161	0.6223	0.3790	0.3781	0.9733
rs7201	C/A	23/109/182	20/174/246	0.1517	0.2468	0.2462	0.8715

*Abbreviations*: *SNP* single nucleotide polymorphism

^a^Alleles 1 and 2 represent the major and minor alleles, respectively, and the genotype counts are shown in the order of 11, 12, and 22, respectively

**Fig. 1:**
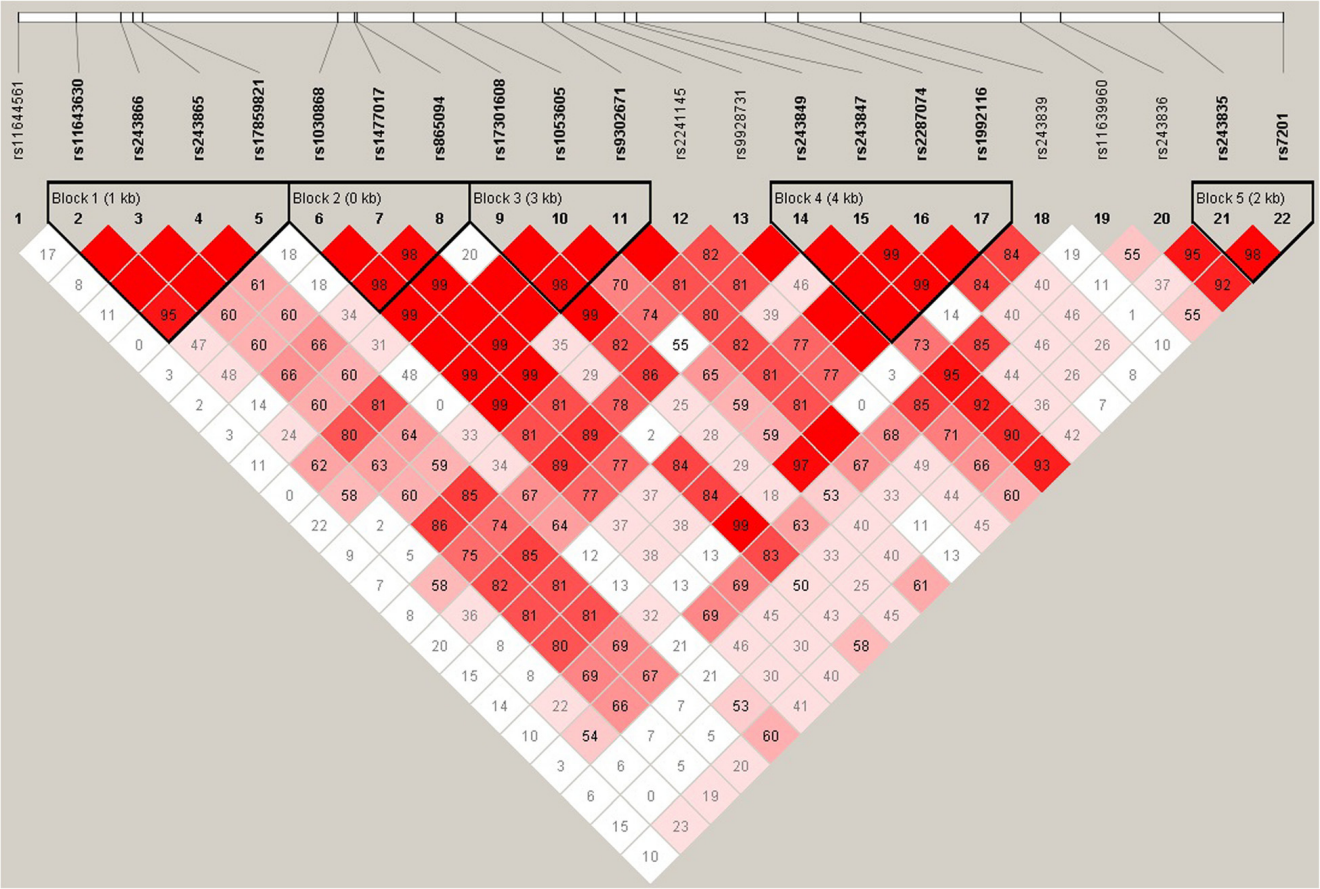
The reference single-nucleotide polymorphism (rs) locations and block structure of MMP-2 in samples from the study population. Pairwise linkage disequilibrium relations between MMP-2 polymorphisms were reported using correlation coefficients (r^2^)

Haplotype analysis was performed to further evaluate the role of MMP-2 in TAD susceptibility. The LD structure was constructed with all SNPs genotyped (Fig. 1). Four haplotype-blocks were defined based on the default algorithm of Gabriel et al. [23] and used for subsequent analyses. Overall, this four haplotype frequencies were similar between cases and controls. No significant omnibus association was detected. Results from the haplotype-based omnibus association analysis showed no evidence for any association with risk of TAD (data not shown).

To further assess haplotype association, we conducted a sliding-window analysis. There were 253 sliding windows in MMP-2 gene. And there were 43 sliding windows showing significant differences (P_emp_ <0.05) in MMP-2 haplotype frequencies between case participants and control participants with at least 1 significant window among sliding windows of a given size with at least 2 SNPs (Table 3). The importance of rs9928731 was obvious because 39 of 43 “positive” windows contained this SNP. The 2-SNP window rs2241145 and rs9928731 (Table 3) gave the most significant result: P_asym_ = 7.48 × 10 ^−5^. It remained significant after 15,000 permutations correcting for multiple testing (P_emp_ = 0.001867). Of the 43 positive sliding windows, 38 windows carried these 2 SNPs. Our conditional logistic regression analysis indicated that each of these 2 SNPs contributed independent effects to the significant association between this 2-SNP window and TAD: P = 1.71 × 10 ^−6^ for rs2241145, P = 1.54 × 10 ^−6^ for rs9928731.

**Table 3:** Summary of Exhaustive Haplotype Analyses Based on Omnibus Tests for Sliding Windows of All Possible Sizes Across Separate Sets of Tag SNPs of MMP-2

		No od SWs	Most signifcant omnibus test		
SNPs No	SWa, No	With Pemp < .	SW	Pasym	
		05	PEmp		
1	22	0	rs11644561…rs11644561	0.0579	0.7502
2	21	1	rs2241145…rs9928731	7.48e-005	0.001867
3	20	2	rs9302674…rs9928731	0.00041	0.01153
4	19	3	rs243847…rs243839	0.000176	0.004666
5	18	5	rs243847…rs11639960	0.000223	0.0058
6	17	4	rs9302671…rs2287074	0.00031	0.008399
7	16	2	rs1093605…rs2287074	0.000739	0.022
8	15	2	rs17301608…rs2287074	0.000548	0.0158
9	14	4	rs1053605…rs243839	0.000678	0.0206
10	13	3	rs1053605…rs11639960	0.00039	0.01127
11	12	3	rs65094…rs243839	0.000817	0.02413
12	11	1	rs17859821…rs2287074	0.000834	0.02467
13	10	3	rs865094…rs243836	0.000767	0.02247
14	9	4	rs11643630…rs243847	0.000611	0.0182
15	8	9	rs11643630…rs2287074	0.000147	0.003866
16	7	1	rs11643630…rs19992116	0.000144	0.003866
17	6	0	rs11643630…rs243839	0.00221	0.0658
18	5	0	rs243866…rs243836	0.00395	0.1135
19	4	1	rs11643630…rs243836	0.000914	0.0276
20	3	1	rs11644561…rs243836	0.00129	0.04006
21	2	0	rs11644561…rs243835	0.00177	0.052
22	1	0	rs11644561…rs7201	0.00175	0.05173

*Abbreviations*: *P*_*asym*_ asymptotic P value; *P*_*emp*_empirical P value, *SNP* single nucleotide polymorphism, *SW* sliding window

There were 2 protective haplotypes: CT (21; OR, 0.403) and GC (12; OR, 0.448), for which alleles 1 and 2 stand for the major and the minor alleles, respectively (Table 4). For the CT haplotype, the frequencies were about 2.1 % in the case group and more than 5.8 % in the control group. For the GC haplotype, the frequencies were about 3.5 % in the case group and 8.2 % in the control group. We further evaluated effects of genetic models for the risk haplotype defined by the 2 SNPs rs2241145-rs9928731. We found that the 2-SNP haplotype window rs2241145-rs9928731 in MMP-2 gene also showed significant association with TAD in both additive and dominant models (P = 0.002; OR, 0.6).

**Table 4:** Data on 2-SNP haplotypes consisting of rs2241145 and rs9928731

	Frequency			
Haplotype	Cases	Controls	OR	Pasym
GT(11)	0.4523	0.4019	1.23	0.0527
CT(21)	0.0205	0.0583	0.403	0.00303
GC(12)	0.0349	0.0822	0.448	0.000976
CC(22)	0.4923	0.4576	1.15	0.189

*Abbreviations*: *OR* odds ratio; *P*_*asym*_ asymptotic P value

These results were based on analysis that did not take cardiovascular risk factors as covariates into account. To account for the interference of potential confounding factors with our assessment of the relationship between the haplotypes and TAD, we performed a multivariate logistic regression analysis that was adjusted for the covariates such as age, gender, hypertension, diabetes, dyslipidemia, coronary artery diseases, alcohol and smoking habit. When these cardiovascular risk factors were included as covariates in the analysis, the conclusion remained the same, with slight variation in the actual P values and ORs (data not shown).

Consistent with others’ observations [24, 25], there were significantly more male TAD patients than female TAD patients in this study. In order to minimize the influence of gender difference and detect possible interaction between gender and haplotype frequencies, we calculated association with TAD in gender-specific case–control analysis. We verified the association of the 2-SNP haplotype window rs2241145-rs9928731 in MMP-2 gene to the risk of TAD both in subsamples of males and females: P = 0.0005 for males, P = 0.0088 for females. (Table 5).

**Table 5:** Data on 2-SNP Haplotypes Consisting of rs2241145 and rs9928731 in the gender-stratified subsamples

Group	Haplotype frequency				Pasym
	GT	CT	GC	CC	
Males					
TAD cases	0.4538	0.0253	0.0337	0.4872	0.0005
Controls	0.4039	0.0515	0.0894	0.7552	
Females					
TAD cases	0.4481	0.0046	0.0384	0.5089	0.0088
Controls	0.3997	0.0656	0.0749	0.4599	

*Abbreviation*: *P*_*asym*_asymptotic P value

## Discussion

A comprehensive way to examine gene is through a tag SNP approach [26–28]. In the current study, by using a SNP tagging approach, we identified and evaluated 22 tag SNPs that efficiently encompasses most of the common variants in the MMP-2 gene. To our knowledge, this is the first study to use such a comprehensive approach to investigate the roles of the MMP-2 gene in thoracic aortic dissection. Our initial single marker analysis did not find any significant difference in the genotypic distribution and allele frequencies between the TAD patients and controls for all 22 tag SNPs selected after Bonferroni correction. (Table 1). Furthermore, since the haplotype-based association method may be more powerful than the single locus test for indirect LD association mapping [29–31], we analyzed haplotype association on these SNPs. Haplotype blocks were defined according to Gabriel et al. in Haploview software [23, 32]. We also adopted a variable-sized sliding-window strategy to evaluate the haplotypic effects thoroughly. 43 of 253 possible sliding windows in the MMP-2 gene showed significant differences in haplotype frequencies between case participants and control participants (Table 2). The 2-SNP window rs2241145-rs9928731 showed the strongest association with risk of TAD and the two constituent SNPs each contributed independent effects to the haplotypic association. There were two protective haplotype defined by these 2 SNPs (Table 4).

MMP-2 has been considered as a target candidate gene of genetic association studies for numerous human diseases [33, 34]. As was mentioned in the introduction, an association between MMP-2 SNPs and some tissue remodeling-related diseases was recently reported in several studies [14–16]. Several high-interest SNPs that have been reported in these literature were also chosen. However, we failed to find any significant difference between the TAD cases and controls with these SNPs. There may be several reasons for this discrepancy. First, this might be explained by the fact that the etiology and pathogenesis of TAD is different compared to the other tissue remodeling-related diseases. For example, a thrombus can lead to an embolic stroke [35], which is different from TAD formation. Second, determining what causes these diseases is complex. It is not a surprise since it is common in complex diseases that the significant association signals in one investigation appear to be negative in other analyses. Behavioral and environmental factors may be involved in the pathogenesis of these diseases. Third, both different genetic backgrounds between populations and minor diversities in sample collection could contribute to the discrepancy between the studies.

In the current study, we failed to find any significant difference with our initial single marker analysis after correction for multiple testing. However as we mentioned above, haplotype-based association method may be more powerful than the single locus test for indirect LD association mapping [29–31]. We verified that the two constituent SNPs rs2241145-rs9928731 haplotype window contributed to the risk of TAD. The inconsistency of data observed between single marker analysis and haplotype analysis might indicate that not one SNP independently associated with risk of TAD, but the two SNPs in combination. Also these two SNPs themselves might not be the functional SNP. It was the gene region related to this two constituent SNPs rs2241145-rs9928731 haplotype which carried functional aetiological variants.

For the 22 tag SNPs under our study, the most important one is rs9928731 as 39 of 43 “positive” sliding windows contained this SNP. It was previously reported to be associated with refractive error [36] and high myopia [37]. During myopia development, the sclera undergoes active remodeling, which involves MMP-2 [38]. However it still has to be identified whether this variation is the functional SNP causing these risk. This variation is located in the intron of MMP-2 which is between the sixth and seventh exons. This region does not contain any obvious regulatory elements which could predict that rs9928731 is a functional variant. It is also possible that the functional SNP is not rs9928731, but a SNP in linkage disequilibrium with the true causal variants. Clearly, further studies designed to study the mechanistic function of this polymorphism and its clinical applications are needed.

We should recognize the limitation of this study because of the gender difference between patients and controls. In order to minimize the influence of gender difference and detect possible interaction between gender and SNP genotype status, we calculated associations with TAD in gender-specific case–control analysis. We also utilized multivariate logistic regression analysis to adjust effects of clinical covariates including g`ender. Based on the consistent significant findings of the 2-SNP haplotype window from gender-stratified analysis and logistic regression analysis, we are confident that the risk estimation we report is valid.

## Conclusion

In conclusion, we have taken a tag SNP approach to assess the role of common variation throughout the MMP-2 gene for an association with thoracic aortic dissection. With a sliding-window analysis, we found that the MMP-2 2-SNP window rs2241145-rs9928731 showed the most significant association with TAD. Therefore, MMP-2 polymorphisms are likely to play an important role in the genetic predisposition to TAD. These findings potentially may have significant benefit for the individualized treatment of TAD in the future.

### Availability of data and materials

The datasets supporting the conclusions of this article are included within the article and its additional file 1.

## Additional file

Additional file 1:
**The dataset supporting the conclusions of this article.** (XLSX 106 kb)

## Abbreviations

TAD: Thoracic Aortic dissection
MMP-2: Matrix metalloproteinase-2
ECM: Extracellular matrix
SNP: Single nucleotide polymorphisms
EDTA: Ethylenediamine tetraacetic acid
MAF: Minor allele frequency
MALDI-TOF: Matrix-assisted laser desorption ionization time-of-flight
HWE: Hardy-Weinberg equilibrium
LD: Linkage disequilibrium
